# Efficacy of Hijamat (wet cupping therapy) in Iranian patients with nonalcoholic fatty liver disease: a controlled clinical trial

**DOI:** 10.3906/sag-1907-82

**Published:** 2020-04-09

**Authors:** Homayoon BASHIRI, Arezoo BOZORGOMID, Vahid SHOJAEIMOTLAGH

**Affiliations:** 1 Infectious Diseases Research Center, Kermanshah University of Medical Sciences, Kermanshah Iran; 2 Department of Internal Medicine, Kermanshah University of Medical Sciences, Kermanshah Iran; 3 Department of Medical Surgical Nursing, Khoy University of Medical Sciences, Khoy Iran

**Keywords:** Nonalcoholic fatty liver disease, wet cupping, Hijamat, ALT, AST

## Abstract

**Background/aim:**

Nonalcoholic fatty liver disease (NAFLD) is known to be the most prevalent chronic liver disease all over the world. The incidence of this disease has dramatically increased during the last decade. Studies have shown a strong relationship between the level of ferritin and the severity of NAFLD. The objective of the present study was to assess the effect of adding Hijamat, as an iron reducing procedure, to standard lifestyle modiﬁcation on the improvement of insulin resistance and liver enzymes in patients with NAFLD.

**Materials and methods:**

One hundred and twenty NAFLD patients participated in a randomized, controlled, single-blind trial design of study. The control group received counselling for nutrition and physical activity for a period of 6 months. The treatment group received the above items plus Hijamat for 3 times during 1 month. Ultrasound images of liver, HOMA-IR, and laboratory data including ALT, AST were assessed pre- and postintervention.

**Results:**

At the end of the study, a signiﬁcant decrease was demonstrated in the serum level of HOMA-IR (–1.30 ± 0.88 vs –.02 ± 0.47, P < 0.001) and serum levels of ALT (–6.50 ± 4.92 vs –2.38 ± 3.92, P < 0.001) and AST (–2.78 ± 4.29 vs –1.30 ± 2.33, P = 0.021) in the Hijamat group were compared to the control group. Ultrasound images of the liver improved in 23.3% of the patients in the Hijamat group, while the rate of improvement in the control group was 10% (P = 0.050). Hijamat therapy was safe and tolerable in this trial.

**Conclusions:**

Hijamat resulted in a relative improvement in fatty liver severity and improved HOMA-IR and liver enzymes more than lifestyle modification alone in patients with NAFLD.

## 1. Introduction

Nonalcoholic fatty liver disease (NAFLD) represents a spectrum of diseases ranging from steatosis to steatohepatitis, fibrosis, cirrhosis and even hepatocellular carcinoma which is characterized by lipid accumulation within hepatocytes [1]. The incidence of NAFLD has been increasing during the last 3 decades, reaching 6%–35% in the adult population worldwide [2]. One of the most important reasons for this high prevalence is the globalization of sedentary lifestyle and modern dietary habits together with increased prevalence of central (abdominal) obesity, type 2 diabetes mellitus, dyslipidaemia and metabolic syndrome [3]. 

Although the molecular mechanisms involved in the pathogenesis of NAFLD are still poorly understood, insulin resistance contributes to increased delivery of free fatty acids to the liver and overproduction of lipids in the liver, eventually leading to NAFLD [4]. According to various studies, high levels of serum ferritin, as a standard marker of iron storage are associated with an increase in free radicals and induce insulin resistance in myocytes, adipose tissue [5].

Wet cupping therapy or Hijamat is one of the oldest medical techniques in Asia, the Middle East and Europe. It is widely referred to Iranian traditional medicine documents for prevention and treatment of various disorders [6]. In this procedure, causative pathological substances are excreted from the interstitial ﬂuid and blood in the skin capillary network after sucking and scariﬁcation steps [7]. A few studies have suggested that removal of iron by phlebotomy leads to improved insulin resistance and liver enzymes in NAFLD patients with normal serum ferritin and transferrin levels [8,9]. Unlike Hijamat, one of the superficial veins of the body is cut with a scalpel and some blood is removed from the body in phlebotomy [10]. Most people usually prefer Hijamat to phlebotomy because it is easier and causes less pain. In light of the cumulative evidence for the association between serum ferritin and NAFLD, this study was conducted to evaluate the role of Hijamat therapy in the improvement of liver enzymes and HOMA-IR in patients with NAFLD.

## 2. Materials and methods

### 2.1. Study design 

The study was a randomized, single-blind (observer-blind) trial and the duration of the study was 6 months. The study design is depicted in Figure. The patients were randomly divided to the treatment and control groups using the stratified blocked randomization method with a computer. All NAFLD cases initially received counselling to reduce the intake of carbohydrate, red meat, fried foods, sugars, and fat and increase fibre intake and physical exercise (at least 20 min, 3 times per week) as a routine treatment. After 4 months, the treatment group received Hijamat for 3 times during 1 month (0 days, 14 days, and 28 days) [11]. Hijamat was performed using sterile disposable cups at 5 points: both scapulas, both lumbar regions and vertex. Disposable cups were placed on these points and negative pressure was applied by a cupping pump. The cups were removed after about 2–3 min. Then, 26-gauge disposable lancets were used for scarification to a depth of 2 mm at sites of cups. Vacuum pumping was repeated for the second time and 3–5 cm3 of a bloody excretion was removed per cupping site. The sites were then covered with sterile pads.

### 2.2. Study participants

Subjects were selected from those adults aged 18 years or above consecutively referred to the Gastroenterology and Hepatology Clinic of the Imam Reza Therapeutic Educational Hospital (Kermanshah, Iran) with a diagnosis of NAFLD. This hospital is the largest centre among referral centres in Kermanshah Province, west of Iran. A diagnosis of NAFLD was made according to the clinical evidence, liver enzymes and ultrasonography criteria. All subjects provided informed written consent for the study. The patients with viral hepatitis B and/or C infection, jaundice, autoimmune hepatitis, Wilson’s disease, α-1-antitrypsin deficiency, type 1 or secondary forms of diabetes, celiac disease, alcohol consumption, corticosteroids consumption and kidney disorders as well as pregnant or lactating women were excluded from the study. Demographic data such as age, sex and medical history were gathered from patient interviews during screening. 

**Figure 1:**
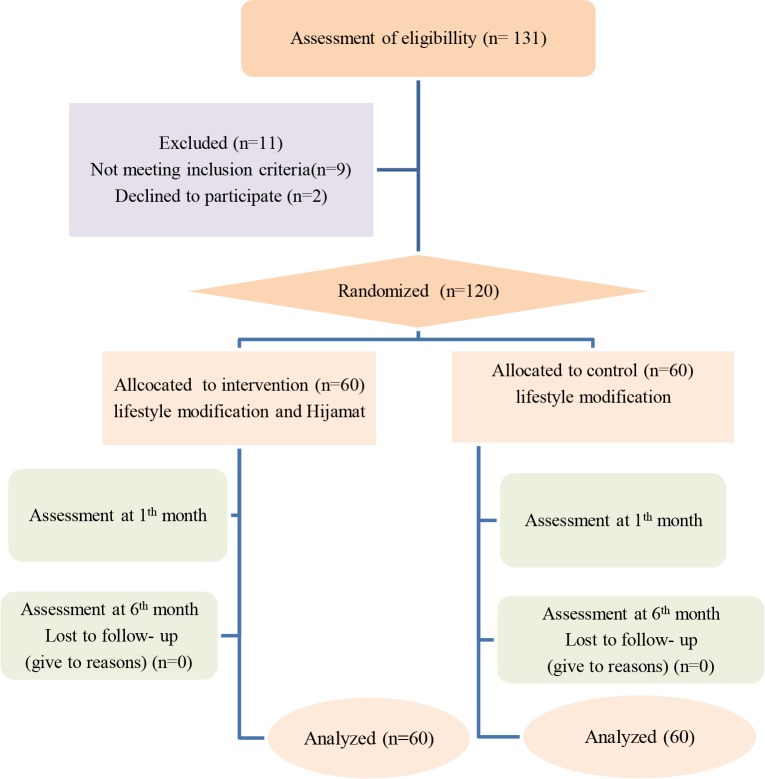
Flow chart of the allocation, follow-up, and analysis of this clinical trial.

### 2.3. Sample size

There were no similar studies to help us with the study sample size. It has been recommended that around 60 patients are a reasonable sample size for a pilot study to investigate the treatment effects [12]. Thus, we decided to recruit at least 60 patients in each group as a convenience sample. 

### 2.4. Biochemical assessment

Blood samples were collected from participants after a 12-h overnight fast before and after the intervention. After serum separation, the samples were labelled and stored at –70 °C until analysis. Enzymatic activities of alanine aminotransferase (ALT) and aspartate aminotransferase (AST) were measured with the Hitachi 902 autoanalyzer (Japan) using Pars Azmoon (Iran) analytical kits. Laboratory reference ranges for ALT and AST were 5-41 IU/L and 5-37 IU/L, respectively. Insulin resistance was determined using the homeostasis model assessment (HOMA-IR) by the following formula: fasting insulin (µu/mL) × fasting glucose (mmoL/dL)/405. 

### 2.5. Ultrasound imaging of the liver 

Ultrasound images of each participant were also evaluated before and after the intervention. Hepatic steatosis was graded as 0 (no fat accumulation), 1 (minimal increase in echogenicity along with normal appearance of the diaphragm and portal vein wall), 2 (moderate increase in echogenicity along with slightly impaired visualization of the portal vein wall and diaphragm), and 3 (severe increase in echogenicity along with poor or no visualization of the diaphragm, portal vein wall and the posterior portion of the right hepatic lobe) [13]. 

### 2.6. Statistical analysis

The SPSS software was used for statistical analysis version 16 (SPSS Inc., Chicago, IL, USA). The Kolmogorov–Smirnov test was applied to evaluate the normal distribution of the data in each group. Continuous variables are presented as mean ± standard deviation (SD). Independent-samples t-test or Mann–Whitney U test was used for between-group comparisons as appropriate and paired t-test was used for within-group comparisons. Categorical variables were compared using chi-square test. The P < 0.05 was considered as statistically significant.

## 3. Results

As shown in Figure, 11 of 131 assessed patients with NAFLD were excluded from the study for various reasons. A total of 120 patients (61 males, 59 females) with mean age of 39.46 ± 8.38 were assessed in this study. The patients were randomly divided into 2 groups, including 60 patients in the treatment group and 60 subjects in the control group. The mean age of the subjects in the treatment and control group was 38.38 and 40.53 years, respectively (P = 0.161). There were 30 female participants in the treatment and 29 in the control group. (P = 0.855). There were no statistical diﬀerences between groups for other baseline characteristics (P > 0.05), so the groups were comparable. The patients’ characteristics are described in Table 1.

**Table 1:** Baseline characteristics and biochemical parameters of control and Hijamat groups.

Variables	Control group (n = 60)	Hijamat group (n = 60)	P-value
Age, years	40.53 ± 7.32	38.38 ± 9.26	0.161^a^
Gender, female (%)	29 (48.33)	30 (50)	0.855^b^
ALT, IU/L	46.30 ± 4.26	45.88 ± 4.39	0.883^c^
AST, IU/L	45.46 ± 5.25	44.33 ± 4.79	0.220^a^
HOMA-IR	2.23 ± 0.62	3.19 ± 0.65	0.630^c^
Sonography (%)			
Grade I	26 (43.3)	31 (51.7)	0.364^b^
Grade II	34 (56.7)	28 (46.7)	
Grade III	-	1 (1.7)	

Values are expressed as mean ± SD

^a^Independent sample t-test

^b^Chi-square test

^c^Mann–Whitney U test

A paired t‑test showed that after 6 months of Hijamat, mean ALT, AST and HOMA-IR decreased significantly from baseline (P < 0.001). In the control group, all variables after study were significantly decreased compare to the baseline except for HOMA-IR (P = 0.724) (Table 2).

**Table 2:** Comparison between baseline laboratory parameters and after 6 months of intervention.

Variables	Control group			Hijamat group		
	Baseline	After 6 months	P-value
Baseline	After 6 months	P-value	ALT, IU/L	46.30 ± 4.26	43.91 ± 3.70	<0.001
45.88 ± 4.39	39.38 ± 4.22	<0.001	AST, IU/L	45.46 ± 5.25	44.17 ± 5.74	<0.001
44.33 ± 4.79	41.55 ± 4.43	<0.001	HOMA-IR	2.23 ± 0.62	2.21 ± 0.71	0.724
3.19 ± 0.65	1.89 ± 0.52	<0.001

Data are Mean ±SD.

P values calculated by paired sample t-test

According to the data shown in Table 3, signiﬁcant improvements were observed in HOMA-IR and serum levels of ALT and AST in the treatment group compared to the control group after the intervention (P < 0.05). Hijamat was safe and tolerable in this trial and there were no reports of any side effects. The ultrasonographic findings of the liver improved significantly in the case group compared to the control group (P < 0.001). The fatty liver grade improved in 23.3% of the subjects in the Hijamat group, while the rate of improvement was 10% in the control group (Table 4).

**Table 3:** Comparison of mean changes in studied variables of 120 patients with nonalcoholic fatty liver disease.

Variables	Control group	Hijamat group	P-value
Change in ALT, IU/L	–2.38 ± 3.92	–6.50 ± 4.92	<0.001
Change in AST, IU/L	–1.30 ± 2.33	–2.78 ± 4.29	0.021
Change in HOMA-IR	–.02 ± 0.47	–1.30 ± 0.88	<0.001

Data are mean ± SD.

P-values calculated by independent sample t-test.

**Table 4:** Evaluation of fatty infiltration in liver tissue after the intervention between studied groups.

Fatty infiltration	Control group		Hijamat group		P-value
	Frequency	Percent	Frequency	Percent	
Improved	6	10	14	23.3	0.050
Not recovered	54	90	46	76.7	

P-values calculated by chi-square test.

## 4. Discussion

This study was conducted to investigate whether or not Hijamat therapy is effective in the improvement of liver enzymes and histology in patients with NAFLD. To the best of our knowledge, this study is the first published clinical trial of the effect of a combination of Hijamat and lifestyle modification on liver enzymes and HOMA-IR in patients with NAFLD. Therefore, considering the low number of research articles, we are only able to discuss the hypothetical mechanisms of Hijamat that may provide clues to the research question. 

The results showed a signiﬁcant improvement in the serum levels of ALT and AST in the Hijamat group versus controls. Evidence suggests an association between increased serum ferritin and ALT levels [14]. Increased ferritin and body iron stores can lead to advanced fibrosis in patients with NAFLD [15]. Studies have shown that iron chelation therapy and Hijamat could be beneficial in patients with mild iron overload. El-Shanshory et al. (2014) selected 40 thalassaemic children and divided them into 2 groups: 20 subjects received iron chelation therapy (ICT) plus Hijamat and 20 patients received iron chelation therapy as the control group. The authors concluded that Hijamat could significantly decrease iron overload, potentiate ICT and decrease oxidative stress. Therefore, one possible mechanism for therapeutic effects of Hijamat is the excretion of excess iron in the interstitial ﬂuid [16]. 

Furthermore, a signiﬁcantly larger improvement was observed in the HOMA-R index in the case group versus the control group. Hijamat can decrease oxidative stress through decreasing the iron stores, resulting in improved insulin resistance that may lead to decreased production of liver glucose, which is one of the mechanisms for developing steatosis in NAFLD. Moreover, oxidative stress, as a cause of NAFLD, is associated with production of reactive oxygen species [17], either via peroxisomal and mitochondrial β-oxidation of hepatic fatty acids or via inflammatory cell activation. This occurrence leads to increased lipidperoxidation and the release of proinflammatory cytokines, resulting in the impairment of cell membrane integrity [17]. 

Several studies have evaluated the effect of Hijamat on different biochemical markers and found that it was an effective alternative therapy. For example, it has been suggested that Hijamat can effectively reduce the levels of cholesterol and LDL and prevent cardiovascular disease [18]. It is also worth mentioning that there is a significant positive correlation between increased levels of cholesterol and LDL and increasing grades of fatty liver [19]. Mahaling et al*.* found that individuals with fatty liver had increased levels of cholesterol, LDL, and very LDL (VLDL) and decreased levels of HDL [20].

Khodadoostan et al. evaluated the effect of phlebotomy on liver enzymes and histology in patients with NAFLD. The authors concluded that phlebotomy decreased liver cell damage and improved liver enzymes and histology [9]. Another study conducted in Italy evaluated patients with nonalcoholic fatty liver disease and hyperferritinemia. The result showed a significant reduction in insulin resistance following phlebotomy in the treatment group as compared to the control group [21]. Nevertheless, Hijamat may be superior to other excretory procedures such as phlebotomy. Unlike phlebotomy, Hijamat rarely causes anaemia, as blood cells are not ﬁltered through capillary pores. Moreover, it filters and clears interstitial ﬂuids from excess ﬂuids and/or soluble causative pathological substances that were reported to be rich in iron [10]. Hijamat therapy is a very safe procedure when performed by trained health staff. However, few studies have reported some side effects for cupping therapy, such as persistent skin discoloration, bruising, scars, burns and infections [22]. Nevertheless, Hijamat is contraindicated in subjects under 2 and over 60 years of age and in very slim and weak or extremely obese individuals [23].

Although Hijamat was more effective on liver enzymes; the benefits of lifestyle modifications are well demonstrated. Several studies have shown that permanent lifestyle changes induce weight loss and improve liver enzymes and histological outcomes in NAFLD [24,25].

Several points should be considered as the strengths of this study such as its novelty in introducing a safe and affordable therapy for improvement of liver enzymes and histology in patients with NAFLD. However, the study also had several limitations like not assessing many fatty liver biochemical markers such as oxidative stress, serum lipid profile and serum and liver iron levels, a short follow-up period, and lack of daily assessment of calorie intake by patients. Also due to the nature of Hijamat, it is not possible to blind participants to performed intervention. 

Taken together, the results of this study implied that Hijamat was a safe and economic, and the complementary use of Hijamat plus lifestyle modification could be more effective in reducing HOMA-IR and ALT and AST levels and improve the fatty liver grade.

## Acknowledgments/Disclaimers

The authors want to thank their colleagues in Imam Reza Therapeutic Educational hospital of Kermanshah, Iran for their contribution to the patient’s diagnosis. We also extend our thanks to clinical research development center of Imam Reza Hospital for their kind advise. This study was supported by a grant from the Vice Chancellery for Research and Technology, Kermanshah University of Medical Sciences (grant number: 89065).

## Informed Consent

All the participants gave written informed consent prior to the study and this study was conducted in accordance with the Declaration of Helsinki. The protocol was approved by the Ethics Committee of Kermanshah University of Medical Sciences (IRCT138905154520N1).
